# Clinical characteristics and sociodemographic profile of patients with First Bipolar Mania

**DOI:** 10.1192/j.eurpsy.2023.1474

**Published:** 2023-07-19

**Authors:** M. Gardabbou, R. Feki, I. Gassara, N. Smaoui, M. Maalej Bouali, N. Charfi, L. Zouari, J. Ben Thabet, S. Omri, M. Maalej

**Affiliations:** psychiatry C department, Hedi Chaker, Sfax, Tunisia

## Abstract

**Introduction:**

Mania is a serious psychiatric condition, characterized by high rates of relapse and significant dysfunction. An early understanding of the factors involved with the manifestations of this disease is critical as to help estimate impact and plan appropriate treatment modalities.

**Objectives:**

To assess demographic and clinical characteristics in a first bipolar mania and describe the associations between these factors.

**Methods:**

A retrospective study was carried out for descriptive and analytical purposes targeting the demographic and clinical characteristics of patients admitted for the first time for a mania within the psychiatry « C » department of Sfax, Tunisia between 2019 and 2022.

**Results:**

Our study included 50 male inpatients, with a median age of 31.8 years (min=18, max=62) at the moment of their hospitalisation. One third of the patients was married. Only 18% got postsecondary education and 65.3% had a profession. A total of 26.5% had a low socioeconomic status. Twenty-four percent suffered from substance abuse and 14% had a criminal record. Personal psychiatric history was noted in 32% of cases and a personal medical history in 16% of cases. Psychotic features were found in 89.8% of patients. Heteroaggressiveness was present in three quarters of cases, whereas an expansive mood was found in half the population. Twenty percent of patients had a poor insight. A statistically significant association was found between being employed and the absence of personal psychiatric history (p=0.017), whereas personal medical history was associated with a poor insight (p=0.049). Substance abuse was correlated with having a criminal record (p=0.006) and heteroagressiveness (p=0.012). The presence of psychotic features was positively associated with expansive mood (p=0.022).

**Conclusions:**

This study confirms that some epidemiological factors are strongly associated with clinical characteristics of the bipolar mania. Early interventions over these factors may contribute to a potential reduction in morbidity and mortality of this disease.

**Disclosure of Interest:**

None Declared

